# Fatty Acid Composition of Total Lipids in Atlantic Salmon *Salmo salar* L. Parr and Smolts Reared in Aquaculture at Various Lighting Regimes

**DOI:** 10.1134/S0012496623700734

**Published:** 2023-11-11

**Authors:** D. S. Provotorov, S. A. Murzina, V. P. Voronin, A.E. Kuritsyn, N. N. Nemova

**Affiliations:** Institute of Biology, Karelian Research Center, Russian Academy of Sciences, Petrozavodsk, Russia

**Keywords:** fatty acids, ontogeny, adaptation, light, feeding, aquaculture

## Abstract

Continuous artificial lighting (24LD) was introduced experimentally in the standard technology to grow salmon juveniles in a southern region (Republic of North Ossetia–Alania) and its effect on fatty acid (FA) composition of total lipids in muscles and the liver was assessed in parr and smolts of the Atlantic salmon *Salmo salar* L. Changes in the key FA were observed in the FA spectrum of fish, indicating that smoltification was complete and that smolts were ready for new habitat conditions. Thus the content of polyunsaturated FAs (PUFAs) significantly increased as a result of an increase in (*n*-3) PUFAs, and, in particular, marine-type 22:6(*n*-3), and high values were observed for (*n*-3)/(*n*-6) and 22:6(*n*-3)/18:3(*n*-3) PUFA ratios. The most significant changes were detected in muscles. In all experimental groups, a decrease in saturated FAs (SFAs) and an increase in total lipid unsaturation was attributed primarily to PUFAs, while monounsaturated FAs (MUFAs) decreased along with SFAs. The experimental data on the lipid and FA composition in salmon juveniles and a higher proportion of smolts in the test groups indicated that smoltification was the most successful in groups with continuous lighting and 24-h feeding and a natural regime of lighting and feeding.

## INTRODUCTION

The Atlantic salmon *Salmo salar* is among the most valuable species in aquaculture. Smoltification is an important stage of its life cycle and is associated with a number of morphological, physiological, biochemical, and behavioral modifications in juveniles [1, 2] to allow them to change their habitat from freshwater to seawater. Several factors affect smoltification, and the lighting regime is one of these. Changes in lighting period are known to stimulate the smoltification-related processes, given that the so-called winter (short)-photoperiod regimes are used, i.e., photoperiod regimes are alternated in a summer–winter–summer order with a shorter winter lighting duration (a winter window) [3–5].

Fatty acids (FAs) are accumulated and modified during the growth and development of organisms and respond quickly to changes in environmental conditions, including the lighting regime. In this work, continuous artificial lighting was introduced experimentally in the standard technology to grow salmon juveniles in a southern region (Republic of North Ossetia–Alania). We have previously studied how the lipid status of salmon juveniles (parr and smolts) changes in response to a joint effect of different photoperiods and different feeding regimes in summer and autumn [6, 7]. A 24-h light day (24LD) was observed to ensure a greater effect and to stimulate the preparation to smoltification in Atlantic salmon yearlings. To supplement the previous data, we studied how the FA status changed in salmon parr and smolts changed during smoltification when a regime with continuous lighting and continuous feeding is introduced.

## MATERIALS AND METHODS

The effects of the photoperiod on the growth and development of Atlantic salmon juveniles was studied at the “Ostrov” fish farm (Republic of North Ossetia–Alania). The conditions and specifics of the experiment have been detailed in our previous works [5, 6]. In brief, to stimulate the endogenous mechanisms that affect the growth-related processes, 24LD with LEDs (36 W, 6500 K) was introduced for all tanks with youth of the year (after the change for exogenous feeding). In August, yearlings with an average body weight of 2.3 g were transferred into raising tanks (4 × 1.2 m, 2.5–2.7 m^3^) at 4900 fish per tank. In early September, the fish were divided into three groups, each including two tanks. Experimental conditions were as follows: group 24LD + CF, continuous lighting and continuous (24-h) feeding; group NatLD + DF, natural lighting and feeding every 2 h during daylight hours (from 6:00 a.m. to 6:00 p.m. in September, from 8:00 a.m. to 6:00 p.m. in October, and from 8:00  a.m. to 5:00 p.m. in November); and group 24LD + DF, continuous lighting and daytime feeding (DF), as in group NatLD + DF.

In early December, the young fish were transferred by group into round 2.1-m^3^ tanks (diameter 2 m, height 1 m) at 2800 per tank on average. Additional lighting was not used from December to January according to the above winter window strategy. Further rearing of parr and smolts was carried out in natural illumination conditions of the region. The light intensity at day was 5500 lx (500 lx in cloudy weather).

Commercial feeds were used in the experiment, including Scretting Nutra HP (Italy) fraction 1.8 (from November to February) and BioMar Efico Alpha 790 (Denmark) fraction 3 (from February to March). The feeds were similar in composition and nutritional qualities. Feed amounts were calculated from the amount recommended for the age and the biomass. Losses in the period from December to March were 26, 42, and 34% of fish in groups 24LD + CF, NatLD + DF, and 24LD + DF, respectively.

The average total fish weights (parr with smolts together) on March 2 were 59.44 ± 3.45, 57.71 ± 5.22, and 53.88 ± 4.52 in groups 24LD + CF, NatLD + DF, and 24LD + DF, respectively. The proportions of smolts on March 3 were up to 50% in group 24LD + CF, 40% in group NatLD + DF, and 25% in group 24LD + DF. Salmon parr and smolts were sampled in early March for the study (fish were taken from each group that had additionally been exposed to light during yearling development).

The FA composition of total lipids in muscles and the liver was studied in fish individually. The contents of particular FAs and their proportions were determined by gas chromatography [6].

Tests were carried out at the Laboratory of Environmental Biochemistry and the Collective Use Center of the Karelian Research Center (Russian Academy of Sciences).

Statistical analyzes were carried out using the R programming language (v. 3.6.1) and the functions readxl (v. 1.3.1), tidyverse (v. 1.3.0), ggplot2 (v. 3.4.0), and pheatmap (v. 1.0.12) in RStudio. Particular lipid classes and total FA families were characterized using descriptive statistics (arithmetic mean and error of the mean), which were calculated by month and experimental lighting type. Differences in parameters between parr and smolts were considered significant at *p* ≤ 0.05.

## RESULTS AND DISCUSSION

### Muscle FA Profile in Atlantic Salmon Parr and Smolts

Polyunsaturated FAs (PUFAs) predominated in muscles of parr and smolts. Their proportions in parr were 43.24% in group 24LD + CF, 35.07% in group NatLD + DF, and 38.12% in group 24LD + DF. The proportions in smolts were 47.95, 46.02, and 49.08%, respectively. NatLD + DF, and 24LD + DF Total PUFA predominance was determined by (*n*-3) PUFAs, the proportions of which were 28.53–34.91% in parr and 40.0–42.72% in smolts. Among (*n*-3) PUFAs, docosahexaenoic acid (DHA) occurred at a high proportion, up to 17.87% in parr and up to 30.85% in smolts. The DHA content in smolts was higher than in parr in all experimental groups; the difference was significant in group NatLD + DF. Between-group differences in FA profile were nonsignificant in both parr and smolts.

The proportion of (*n*-6) PUFAs was substantially lower, ranging from 5.67 to 8.33% in part and smolts. Smolts did not display a significant trend to changes in (*n*-6) PUFAs in all of the experimental groups. Predominance in proportion was observed for linoleic acid 18:2(*n*-6) (3.79–6.48%), which occurred in smolts at a significantly lower proportion than in parr (up to 3.84%) in the NatLD + DF group, and arachidonic acid 20:4(*n*-6) (up to 1.51%), which significantly increased in proportion (up to 1.51%) in smolts of the same group. The 20:4(*n*-6)/18:2(*n*-6) ratio, which is the ratio between the product and its precursor utilized in consecutive reactions involving (*n*-6) PUFAs and reflects the efficiency of 18:2(*n*-6) → 20:4(*n*-6) conversion, was significantly higher in smolts of the NatLD + DF group.

The saturated FA (SFA) proportion ranged from 27.12 to 33.74% in fish, and palmitic acid 16:0 was the most prevalent (16.3–31.11%). The SFA proportions did not significantly differ between the experimental groups of parr and smolts, although the SFA content was found to be lower in smolts.

The monounsaturated FA (MUFA) proportion ranged from 22.33 to 31.19% in juveniles (parr and smolts) and tended to be lower in smolts than in parr in all of the experimental group (the difference was significant only in group 24LD + DF). A decrease in MUFAs was mostly due to a decrease in oleic acid 18:1(*n*-9), which was a predominant MUFA. Its proportions were 17.8–19.12% in parr and 13.5–14.88% in smolts. As for other MUFAs, considerable contents were observed for 18:1(*n*-7) (2.63–3.54%), 16:1(*n*-7) (1.77–2.72%), and 20:1(*n*-9) (1.52–2.3%).

### Liver FA Profile in Atlantic Salmon Parr and Smolts

Like in muscles, PUFAs predominated (44.39–46.3%) in the FA profile of the liver in parr and smolts. Significant changes or differences between the experimental groups were not found in parr and smolts. The highest proportion was observed for (*n*-3) PUFAs (30.36–36.18%). Significant dynamics was detected only in smolts of group 24LD + DF (a decrease from 34.58 to 30.36%). The major component DHA did not significantly change from parr to smolts (22.3–27.73%).

The proportions of (*n*-6) PUFAs ranged from 10.12 to 14.03% and increased significantly from parr to smolts (from 10.91 to 14.04%) only in group 24LD + DF. PUFA 18:2(*n*-6) predominated (5.42–8.39%); its proportion in the liver significantly increased from 6.22 to 8.39% from parr to smolts in the same group.

**Fig. 1.**
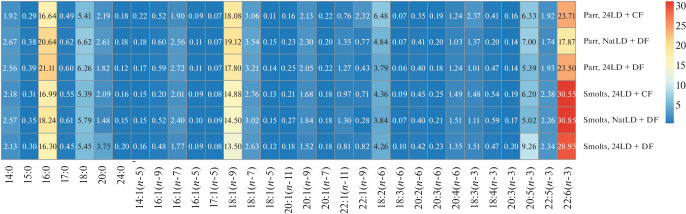
Heat map of the proportions of particular FAs (% in total FA) in muscles of Atlantic salmon juveniles of the three experimental groups in March.

**Fig. 2.**
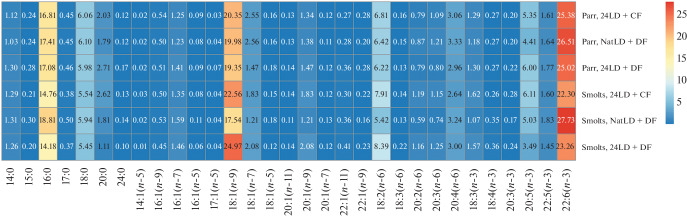
Heat map of the proportions of particular FAs (% of total FA) in the liver of Atlantic salmon juveniles of the three experimental groups in March.

The MUFA content in the liver ranged from 24.88 to 32.95% in Atlantic salmon parr and smolts. A significant increase in MUFA content was detected in smolts of group 24LD + DF. Like in muscles, 18:1(*n*-9) predominated (17.54–24.97%). As for the other FAs of the family, considerable contents were observed for 18:1(*n*-7), 20:1(*n*-9), and 16:1(*n*-7) (Fig. 2).

The SFA proportion in the liver ranged from 22.66 to 28.82% in parr and smolts. A significant decrease in SFA proportion (from 27.97 to 22.66%) was observed in smolts of group 24LD + DF. Predominance was shown for 16:0 (14.18–18.81%), which displayed a significant decrease in smolts of group 24LD + DF.

Thus, PUFAs were the most prevalent in muscles and the liver in both parr and smolts in March, after the so-called winter window. This was due to (*n*-3) PUFAs, which are classed with a marine type. The DHA proportion was found to increase in muscles of smolts. Its increase is usually associated with an increase in locomotion, which is characteristic of salmon smolts as well.

It should be noted that an increase in (*n*-3) PUFAs in muscles was accompanied by a decrease in MUFAs and SFAs in fish of all experimental groups. These findings indicate that lipid metabolism is transformed in salmon during smoltification, as has been demonstrated earlier. Note that group 24LD + CF has displayed the most distinct trend to a higher (*n*-3) PUFA proportion in the first part of the experiment [6]. Generally, group 24LD + CF showed the most distinct and stable trends to preparation of lipid metabolism and its modification from the freshwater to the marine type.

A low proportion (1–2%) of essential 18:3(*n*-3) in muscles and the liver was observed in both parr and smolts. The content of this FA is known to be lower in farmed salmon juveniles, which feed on highly balanced feeds with a sufficient DHA content (marine raw materials), than in wild juveniles, which more intensely accumulate DHA [8]. Lack of essential 18:3(*n*-3) is compensated for by a higher DHA content because DHA and eicosapentaenoic acid (EPA) are known to more efficiently stimulate the muscle growth in fish as compared with 18:3(*n*-3) and to be generally of a higher biological value [9–11]. Another possible factor is that the photoperiod stimulates the enzymatic system of (*n*-3) FA conversion, in which 18:3(*n*-3) is converted through several intermediate products to DHA, as mentioned in our previous work [6]. It should be noted that the (*n*-6) PUFA family showed a substantially lower content as compared with (*n*-3) PUFAs, especially in muscles of fish of group 24LD + CF. The content of arachidonic acid 20:4(*n*-6) in muscles of smolts of all groups was somewhat higher than in yearlings examined in the first part of the experiment [6]. Dietary 18:2(*n*-6), which is the initial FA of the family, is known to accumulate in salmon juveniles reared in aquaculture, while the accumulation of 20:4(*n*-6) is less efficient than in wild juveniles [8, 12]. The liver proportion of arachidonic acid reached values of approximately 3% in parr and remained much the same in smolts. A lower (than in wild fish), but steady accumulation of arachidonic acid may play an important role in adaptation to new environmental conditions because arachidonic acid is a precursor of many biologically active compounds, such as prostaglandins, thromboxanes, and leukotrienes, which are necessary for efficient adaptation in fish. For example, Ca^2+^ influx in the cell is regulated by changes in the amount of prostaglandin F, which acts as an endohormone [13]. Differences in experimental lighting regimes most likely exert no effect on the arachidonic acid content in salmon juveniles.

The changes observed for main FA classes in the liver have some specifics as compared with muscles. In smolts, (*n*-3) PUFAs were similarly the most prevalent in the liver. The FA proportions in total lipids did not significantly differ between groups 24LD + CF and NatLD + DF, indicating that the liver function was physiologically normal and that the lighting and feeding regimes did not affect the liver parameters. DHA, which was the most abundant, showed no significant difference in both parr and smolts. At the same time, significant changes that are not characteristic of smoltification were observed in the liver of fish in group 24LD + CF, i.e., (*n*-3) PUFAs decreased, while (*n*-6) PUFAs and MUFAs increased in proportion. Note that a substantial difference of group 24LD + CF from the two other groups has similarly been observed in our study of the changes in lipid classes in parr and smolts (unpublished). The finding confirms that transformation arises in hepatic lipogenesis in response to 24-h lighting introduced at the yearling stage and is long term, persisting up to the smolt stage (March).

Thus, a regime with 24-h lighting and feeding makes it possible to obtain viable smolts as early as the first year of life when introduced in the technological cycle of salmon youth rearing in aquaculture. The conclusion is supported by the quantitative analyses of the lipid and FA compositions. Key FA parameters were observed to change in the FA spectrum of smolts obtained in the experiment, indicating that smoltification is complete and the smolts are ready to new environmental conditions. Thus the content of PUFAs significantly increased as a result of an increase in (*n*-3) PUFAs, and, in particular, marine-type 22:6(*n*-3), and high values were observed for (*n*-3)/(*n*-6), 18:3(*n*-3)/18:2(*n*-6), and 22:6(*n*-3)/18:3(*n*-3) ratios. The changes were especially pronounced in muscle tissue. A decrease in SAFs and an increase in total lipid unsaturation were primarily due to an increase in PUFAs, while MUFAs decreased along with SFAs in all experimental groups.

Taken together, the experimental data on the lipid and FA composition in salmon juveniles and information about the smolt proportion in the groups, which was obtained from the fish farm, demonstrate that smoltification was the most efficient in groups 24LD + CF and NatLD + DF. Understanding the mechanisms related to changes in FA profile during smoltification will make it possible to optimize the rearing conditions in salmon aquaculture.
